# Familial Idiopathic Intracranial Hypertension in Two Non-obese Chinese Sisters

**DOI:** 10.3389/fneur.2020.569432

**Published:** 2020-11-26

**Authors:** Lei Qiao, Yanping Wei

**Affiliations:** Department of Neurology, Peking Union Medical College Hospital, Chinese Academy of Medical Sciences and Peking Union Medical College, Beijing, China

**Keywords:** familial idiopathic intracranial hypertension, idiopathic intracranial hypertension, diagnosis, headache, papilledema

## Abstract

**Background:** Familial idiopathic intracranial hypertension (FIIH) is a rare condition, the etiology of which is unclear.

**Aims:** To describe two non-obese Chinese sisters who met the criteria of FIIH and to analyze the clinical features and prognosis of FIIH.

**Methods:** The clinical course, treatment, and prognosis of these two patients were analyzed retrospectively. Meanwhile, all the literature of familial IIH (FIIH) was reviewed.

**Results:** These two sisters presented with headaches and visual impairment in their mid-thirties. Magnetic resonance imaging (MRI) of the brain was unremarkable except for partial empty sella. No comorbidities or defined causes were detected. Headaches were partially relieved by dehydrated medicine, whereas the visual impairment persisted.

**Conclusion:** In cases where patients present with headaches, empty sella are found on an MRI, and there is visual impairment with or without papilla edema, intracranial hypertension should be excluded. Furthermore, we should pay more attention to the relatives of those patients with increased intracranial hypertension.

## Introduction

Idiopathic intracranial hypertension (IIH) is a syndrome of elevated intracranial pressure (ICP) in the absence of clinical, laboratory, or radiologic evidence of identified causative factors (1, 2). According to the criteria updated by Friedman et al. (2), IIH is a diagnosis of exclusion which requires the following: (1) papilledema; (2) normal neurologic examination except for cranial nerve abnormalities; (3) neuroimaging that finds normal brain parenchyma without evidence of hydrocephalus, mass, structural or vascular lesions, or meningeal abnormalities; (4) normal CSF composition; and (5) elevated ICP (opening pressure > 25 cmH_2_O in adults).

The etiology of IIH remains unknown, but it is most often seen in women of childbearing age either with obesity or with recent weight gain. In 1969, Buchheit and colleagues were the first to report that IIH can affect more than one family member, which was called familial idiopathic intracranial hypertension (FIIH) (3). Apparently, familial cases of IIH are very rare, and whether genetic or epigenetic factors play a role in the majority of cases of IIH still remains unclear. We report a case of FIIH occurring in two non-obese Chinese sisters at similar ages. All the reported FIIH cases were reviewed to reveal the clinical symptoms, concomitant factors, treatment, and prognosis.

## Case Study

Case1: A 39-year-old woman was admitted to our hospital with a history of headache dating back 3 months. The headaches were occasionally associated with transient binocular visual obscurations. As time went by, the headaches increased in intensity and transient right monocular visual loss lasting for several seconds appeared. She was non-obese with a weight of 55 kg and height of 155 meters (BMI 22.9), without history of menstrual irregularity, and had received no drugs. Neurological examination revealed nothing except for papilla edema found by ophthalmoscopic examinations of the fundus. Visual field examinations revealed a larger blind spot in both eyes. Visual acuity was normal. Fluorescein angiography confirmed papilla edema with dilatation of the optic disc capillaries. A lumbar puncture revealed a recumbent opening pressure of 330 mmH_2_O and the components of cerebrospinal fluid were normal, including cell count and protein and glucose levels. No pathogenic organisms were found in the cerebrospinal fluid. Other laboratory studies, including blood cell counts, erythrocyte sedimentation rate, blood electrolytes, blood glucose, immunoglobulins and complement, serum creatine levels, cholesterol, triglyceride, liver function, C reactive protein, anti-cardiolipid antibody, anti-nuclear antibody, thyroid function, hormone level of parathyroid, and estradiol, were normal. No abnormalities were seen on transcranial doppler. Magnetic resonance imaging (MRI) of the brain and the veins only demonstrated partial empty sella and excluded the stenosis or occlusion of the veins in the brain. As we could not identify any causes, idiopathic intracranial hypertension was proposed. After glycerol were taken, her headaches subsided, but papilledema still existed.

Case 2 is the sister of case 1 who presented with headache, vomiting, blurred vision, and pulsatile tinnitus when she was 33 years old. She was 1.58 meters tall and weighed 54 kg (BMI 21.6). Neurological examination disclosed bilateral papilledema and decreased visual acuity (OD, 0.6; OS, 0.4). A lumbar puncture revealed a higher recumbent opening pressure of 400 mmH_2_O and the component of cerebrospinal fluid was normal. MRI disclosed partial empty sella. Other examinations were the same as case 1 and were also unremarkable. The patient was treated with acetazolamide and glycerol, from which her headache subsided and visual impairment receded partially. Eight years later, the patients complained of numbness of the left face. Ophthalmoscopic examinations revealed atrophy of papilla and loss of acuity which was the same as before. Hyperalgesia of the left face was found on physical examination. Lumbar puncture found that recumbent opening pressure was 260 mmH_2_O.

## Discussion

Two cases of FIIH are described in two Chinese sisters. Both of them shared the following characteristic: symptom onset at childbearing age, non-obese, headache as initial and main symptom, papilledema, normal MRI except neuroimaging empty sella, absence of detectable causes, and a benign course. Other symptoms were found in Case 2, including pulsatile tinnitus, decreased visual acuity, and numbness of the left face. These symptoms were also reported in literature. Concomitant features were not revealed except that menstrual irregularity was recorded in Case 1. Although they are sisters, they had lived in different cities since adulthood, which indicated that common environmental factors contributing to IIH couldn't be detected.

Most cases of IIH are sporadic, although a number of studies have suggested a possible genetic transmission. A systematic analysis of families with IIH may yield useful information. We performed an electronic search in the MEDLINE for all papers on FIIH published in English up to now. A total of 82 patients were identified from 39 pedigrees with a clinical diagnosis of FIH, including 68 females and 14 males (3–14). There was a significant female preponderance. Various combinations were as follows: parent-child relation in 19 families (mother-daughter relation in 15 families, mother-son in three families, father-daughter in one family), siblings in 13 families (including three cases of homozygous twins and two case of heterozygous twins), and first-degree cousins in three families. A parent-child relationship was the most common, especially a mother-daughter relation, followed by siblings.

The total mean age at diagnosis was 27.05 ± 13.50 years, with females diagnosed at 27.82 ± 11.89 years, and males at 23.29 ± 19.72 years. The median age for females and males were 26.5 and 15.5 years, respectively. Of the females, 40 female patients were of childbearing age (20–44 years old), 23 females were younger than 20 years, and five other females were older than 44 years. Those counterparts of male patients were eight, three, and three cases, respectively. Totally, the body habitus of 74 patients was recorded. The ratio of non-obese to obese was 14:51 in females and 8:1 in males. BMI was acquired from seven males and 40 females, the mean values of which were 28.96 ± 14.52 and 37.80 ± 10.83, respectively. It is reported that the incidence of IIH is almost 20-fold higher (19.3/100,000) in women aged 20–44 years who are >20% over their ideal weight, compared with the general population (0.9/100,000) (15). Therefore, childbearing age and obesity predisposes females to FIIH, while they were indistinctive in male patients of FIIH.

In 55 patients for whom imaging results were available, 24 had an empty sella, three had Chiari I malformation, and 28 had normal studies. The values of lumbar puncture opening pressure were recorded in 62 patients, the mean value of which was 365.24 ± 108.98 mmH_2_O. The most common symptoms and signs attributable to elevated ICP were headache, impaired visual acuity, papilledema, diplopia, and tinnitus. In addition, five patients complained of radicular pain of the arms or legs and two patients presented with 6th cranial nerve palsy. As for associated features, the following were listed according to their frequency: affective disorder in seven, asthma in four irregular menstruation and functional uterine bleeding in three, CSF rhinorrhea in two, hypothyroidism in two, and orally taken contraceptives in two. Other comorbidities included positive anticardiolipin antibodies, polycystic ovary syndrome, and tetracycline use, each having one case. Interestingly, affective disorders, especially depression, and asthma were the two most common comorbidities, however, their relation to FIIH was not clear.

Data about therapy and prognosis were identified in 30 patients. Acetazolamide was most frequently used, followed by ventriculoperitoneal shunts and steroids. In 20 patients with acetazolamide therapy, the response group, partial response group, and non-response group were 5, 6, and 9, respectively. These three kinds of responses were, respectively, 0, 3, and 2 in steroid usage. Whereas, in six patients having had shunt operations, five achieved remission, except for one patient who experienced no effect. It seemed that shunt surgery was superior to drug therapy. Generally, the course of disease was variable: stable in eighteen cases, total or partial remission in thirteen, remission and relapse in eleven, and totally blind in three.

Our findings, together with those reported previously, strongly suggest that the higher incidence within families, various types of family relations, and the absence of other contributing risk factors implies the possibility of a genetic factor in FIIH. These patients may have an underlying genetic susceptibility as a predisposing factor for the development of FIIH. It is possible that more detailed studies of familial cases may throw further light on the underlying mechanisms responsible for many cases of this syndrome.

Although FIIH was generally benign, three of the 82 patients became totally blind. First-degree relatives of patients with IIH should be routinely screened for signs and symptoms of IIH and offered lumbar punctures and brain MRIs if necessary. Especially when the patients present headaches, empty sella is found on the MRI, or there is visual impairment with or without papilla edema, intracranial hypertension should be highly suspected. Early diagnosis and shunt operation may improve the prognosis.

## Data Availability Statement

All datasets generated for this study are included in the article/supplementary material.

## Ethics Statement

The studies involving human participants were reviewed and approved by Ethics Committee of PUMCH. The patients/participants provided their written informed consent to participate in this study. Written informed consent was obtained from the individual(s) for the publication of any potentially identifiable images or data included in this article.

## Author Contributions

LQ made substantial contributions to conception and design, acquisition of data, analysis and interpretation of data, and in drafting the manuscript. YW was involved in the interpretation of data and drafting the manuscript, as well as in making critical revision of the manuscript. All authors contributed to the article and approved the submitted version.

## Conflict of Interest

The authors declare that the research was conducted in the absence of any commercial or financial relationships that could be construed as a potential conflict of interest.

## Abbreviations

FIIH: Familial idiopathic intracranial hypertension
MRI: Magnetic resonance imaging
IIH: Idiopathic intracranial hypertension
ICP: Intracranial pressure.
